# Quantification of the dynamics of population heterogeneities in CHO cultures with stably integrated fluorescent markers

**DOI:** 10.1007/s00216-020-02401-5

**Published:** 2020-03-04

**Authors:** Johannes Möller, Marcel Rosenberg, Kristoffer Riecken, Ralf Pörtner, An-Ping Zeng, Uwe Jandt

**Affiliations:** 1 Hamburg University of Technology, Bioprocess and Biosystems Engineering, Denickestr. 15, 21073 Hamburg, Germany; 2 Research Department Cell and Gene Therapy, Department of Stem Cell Transplantation, University Medical Centre (UMC) Hamburg-Eppendorf, Martinistrasse 52, 20246 Hamburg, Germany

**Keywords:** Population heterogeneity, Process variability, LeGO, Clonal stability, Automated flow cytometry

## Abstract

Cell population heterogeneities and their changes in mammalian cell culture processes are still not well characterized. In this study, the formation and dynamics of cell population heterogeneities were investigated with flow cytometry and stably integrated fluorescent markers based on the lentiviral gene ontology (LeGO) vector system. To achieve this, antibody-producing CHO cells were transduced with different LeGO vectors to stably express single or multiple fluorescent proteins. This enables the tracking of the transduced populations and is discussed in two case studies from the field of bioprocess engineering: In case study I, cells were co-transduced to express red, green, and blue fluorescent proteins and the development of sub-populations and expression heterogeneities were investigated in high passage cultivations (total 130 days). The formation of a fast-growing and more productive population was observed with a simultaneous increase in cell density and product titer. In case study II, different preculture growth phases and their influence on the population dynamics were investigated in mixed batch cultures with flow cytometry (offline and automated). Four cell line derivatives, each expressing a different fluorescent protein, were generated and cultivated for different time intervals, corresponding to different growth phases. Mixed cultures were inoculated from them, and changes in the composition of the cell populations were observed during the first 48 h of cultivation with reduced process productivity. In summary, we showed how the dynamics of population heterogeneities can be characterized. This represents a novel approach to investigate the dynamics of cell population heterogeneities under near-physiological conditions with changing productivity in mammalian cell culture processes.

**Electronic supplementary material:**

The online version of this article (10.1007/s00216-020-02401-5) contains supplementary material, which is available to authorized users.

## Introduction

Mammalian cell culture processes with CHO cells are still the primary system for the expression of biopharmaceuticals, such as monoclonal antibodies [1–3]. However, population heterogeneities can significantly affect the bioprocess performance, e.g., with differences in the intraclonal protein expression or metabolically different cell populations [4–8]. It was shown that even in monoclonal CHO cell lines ([9], recently discussed in [10]), population heterogeneities with different phenotypes can spontaneously evolve [4, 11–13], and that cell-cycle-dependent population dynamics with variable productivities and metabolic regulations can be present in cell culture processes [14–17]. The formation and dynamics of physically-derived process heterogeneities are studied intensively in bioprocesses, e.g., evaluating spatial gradients of gas bubbles [18], concentration gradients of substrates [19] and oxygen [20], and pH gradients [21]. However, biologically derived (i.e., intrinsic) heterogeneities at the cell population level are mostly not considered appropriately in the field of bioprocess engineering, and the cell populations are usually treated as isogenic [22]. Potential reasons for intrinsic heterogeneities can be gene segregation during cell division and spontaneous mutations, from single base pairs up to chromosomal rearrangements [23, 24].

To our knowledge, only a few methods (e.g., DNA barcoding [25], single-cell cultivation [22]) are so far available to follow and investigate cellular and population changes in mammalian cell culture processes. To study population heterogeneities, Weber (now Riecken) and co-workers developed a lentiviral gene ontology (LeGO) vector system for the permanent fluorescent staining of mammalian cells [26–28]. The LeGO vectors are stably integrated into the cell genome and the color of each cell and all its daughter cells stays constant, which allows to track their changes using flow cytometry or fluorescence microscopy. On the one hand, the vectors can be used for a cell labeling strategy called RGB-marking, able to label cells in all perceivable colors by stochastic mixing of the basic colors red, green, or blue [26–28]. Therefore, the cells express a highly variable amount of one to three fluorescent proteins, which leads to an individual color for each cell. From that time point on, changes (e.g., the expansion of a clone) become observable almost in real-time. On the other hand, LeGO vectors can be used to label the whole cell cultures with a single, distinct fluorescent color. Then, differently labeled cell cultures can be treated (e.g., different conditions, reagents) and mixed thereafter, leading to easily distinguishable sub-populations due to the expressed fluorescent colors. By this, changes in the composition of the mixed cultures and their interactions and impact on the bioprocess can be investigated in various experimental settings. Among others, this system has so far been applied to follow the fate of neural stem cells in adult mouse brain [29] and tumor progression studies [30–32].

In this study, we analyzed cell population changes in two bioprocess engineering–related case studies with antibody-producing CHO cells, which were transduced with LeGO vectors. In case study I, it was evaluated if cell population heterogeneities originate during high passage cultivations. This was motivated based on previously described changes in long-term cultivations [33, 34] and clonal stability studies [13, 35]. Therefore, CHO DP-12 cells were co-transduced with three lentiviral vectors (RGB-marking), leading to cell populations with stochastically mixed colors (color mixing of the three basic colors red, green, and blue as in every computer monitor or TV screen). The progression of these populations was tracked during 130 days of high passage cultivation.

In case study II, the influence of cell preculture treatment on mixed cell population cultures was assessed to investigate the dynamics of population heterogeneities during bioprocesses. Four differently transduced cell line derivatives of CHO DP-12 cells were first generated with separable fluorescence emissions for the use in flow cytometry, which enables to distinguish the cell line derivatives in mixed cultures. As proof of concept, each cell line derivative was cultivated individually to represent a different growth phase. These individually cultivated cells were pooled together and used for inoculation of mixed shake flask cultures, which were tracked using offline flow cytometry. Automated (online) flow cytometry [17] was used in addition to assess population changes in a bioreactor experiment with the same mixed population.

## Materials and methods

### Parental cell line and media

The antibody-producing cell line CHO DP-12 (clone #1934, ATCC CRL-12445, interleukin-8 antibody) was cultivated in chemically-defined media (TC-42, Xell AG, Germany) supplemented with 0.1 mg l^− 1^ LONG R3 IGF-1 (Sigma-Aldrich, Germany) and 200 nmol l^− 1^ methotrexate (Sigma-Aldrich). No serum or antibiotics were used.

#### Preculture

Cryo-cultures (1 ⋅ 10^7^ cells ml^− 1^) were thawed and cultivated in single-use Erlenmeyer baffled flasks (40 ml working volume, Corning, USA). The incubator (LT-X, Kuhner, Switzerland) was controlled at 37 ^∘^C, 5*%* CO_2_, and 85*%* humidity with a shaking speed of 200 rpm (25 mm shaking diameter). Cell expansion was performed using Erlenmeyer baffled flasks (Corning).

### Lentiviral vectors

Weber et al. [26] introduced the LeGO system, which is a HIV-1 derived, self-inactivating, third-generation lentiviral vector, suitable for the transduction of mammalian cells with multi-color fluorescent markers [26]. They showed that the vectors are an efficient tool to label cells and that the integration into the host cell’s genome is stable [26–29]. This system enables the analysis of population- and clone-dependent fates in vitro and in vivo. Exemplary, the LeGO system has been applied so far to investigate cancer heterogeneity [36, 37] and clonal dynamics with various cell types, such as human-induced pluripotent stem cells [38] or neurons in mouse brains [29].

#### Generation of labeled cell line derivatives

Different LeGO-based cell line derivatives were generated in this study, according to [28]. More information about the vectors used and the available fluorescent colors can be found in [26, 27]. The individual vector maps, sequence data, and protocols are available at http://www.LentiGO-Vectors.de. Lentiviral particles were generated, and parental exponentially growing CHO DP-12 cells were transduced with these particles as described in [28]. The generated cell line derivatives are listed in Table 1.

**Table 1:** Cell line derivatives and corresponding LeGO vectors used in this study, vectors can be obtained from Addgene (corresponding number # in brackets) [26, 27]

Cell line derivative	LeGO vector	Fluorescent protein
Case study I		
	C2 (#27339)	mCherry
CHO DP-12 (RGB)	V2 (#27340)	Venus
	Cer2 (#27338)	Cerulean
Case study II		
CHO DP-12 (Venus)	V2 (#27340)	Venus
CHO DP-12 (mTagBFP)	B2 [38]	mTagBFP
CHO DP-12 (mOrange2)	mOrange2 (#85212)	mOrange2
CHO DP-12 (Cerulean)	Cer2 (#27338)	Cerulean

In case study I, 2 ⋅ 10^5^ cells (in 1 ml medium) were transduced with three RGB vectors (red, green, and blue) simultaneously with a multiplicity of infection (MOI) of three (MOI one for each vector). In theory, all perceivable colors can therefore be present [27]. In case study II, the same preculture was split (0.5 ⋅ 10^5^ cells in 0.5 ml medium each) and the cultures were transduced (MOI three for each preculture) individually to express a different fluorescent color. In both cases, cells were first expanded using multiple T-flasks in a static incubator (37 ^∘^C, 5*%* CO_2_) and later transferred to a shaken single-use Erlenmeyer baffled flask (40 ml, Corning), and stored in a cryobank after expansion.

#### Flow cytometry

Debris was excluded using SSC-A vs. FSC-A and doublets were excluded with FSC-H vs. FSC-A gating prior to the remaining flow cytometry (CytoFlex, Beckman Coulter, USA) assays.

##### Case study I

The fluorescence signal of Cerulean was measured with the 405 nm laser and 525/40 nm filter. mCherry and Venus were quantified with the 488 nm laser and 690/50 nm filter (mCherry) and 585/40 nm filter (Venus). Compensation was applied (Venus—0.15 ⋅mCherry; Cerulean—0.039 ⋅Venus) to reduce cross talk in the used flow cytometry assay. The fluorescence signals were quantified with different intensities due to the used lasers. Normalization of the fluorescence intensities was applied to distribute the cells in a three-dimensional space. Therefore, the intensity was normalized (*I*_normalized_) between a lower (*I*_lower_) and upper (*I*_upper_) boundary, as shown in Eq. 1.

1 $$ I_{\text{normalized}} = \left( \frac{\log{(I_{\text{upper}})}-\log{(I_{\text{measured}}})}{\log{(I_{\text{upper}})}-\log{(I_{\text{lower}})}}\right).  $$

*I*_lower_ was defined to 1 ⋅ 10^3^ and *I*_upper_ individually on the maximal intensity as follows: Cerulean, 2 ⋅ 10^5^; mCherry, 2 ⋅ 10^5^; Venus, 1 ⋅ 10^7^. The population size was defined as the number of positive cells (%) in the respective gate, which were defined to be above the previously mentioned lower intensities. All normalizations and 3D plots were generated in MATLAB 2018a. The excitation and emission spectra are shown in Electronic Supplementary Material (ESM) Fig. S1.

##### Case study II

The measured signals of the four different fluorescent proteins were quantified using the 405 nm laser to measure the intensity of mTagBFP and Cerulean (both 450/45 nm filters). Intensities of Venus and mOrange2 were quantified with the 488 nm laser and 585/42 nm filter. No compensation was applied, and the excitation and emission spectra are shown in ESM Fig. S2.

##### Automated and online flow cytometry

An automated and online flow cytometry (AFC) set-up was previously introduced [17] and also applied in this study. In brief, a flow cytometer (Beckman Coulter, Germany) was connected to the bioreactor, and samples were drawn automatically every hour. Flow cytometry analysis of the samples was performed directly, and no washing or staining steps were involved. The total cell number (*X*_t_) was determined with gating, and the viable cell density (*X*_v_) was calculated from offline samples and re-iterated on the online data. More information about the AFC set-up can be found in ESM and in [17].

### High passage cultivation (case study I)

The high passage cultivation was carried out in three parallel single-use Erlenmeyer baffled flasks (40 ml, Corning) as repeated-batch cultivationvs. Initially, each flask was inoculated with 0.3 ⋅ 10^6^ cells ml^− 1^, and the cell densities, viability, and fluorescence intensities were determined every 5 days. The supernatant was frozen, and the required number of cells was passaged into a new flask (0.3 ⋅ 10^6^ cells ml^− 1^). The remaining cells were discarded. The incubator and medium were the same as in “Preculture”. The results presented in this work refer to a cultivation period of 130 days.

### Mixed cultures (case study II)

During the investigation of preculture treatment on mixed cultures, individual cell line derivatives (see Table 1) were cultivated for different lengths of time. Therefore, one of the four cell line derivatives was thawed every 24 h and was cultivated as described above (Preculture). CHO DP-12 (Venus) cells were thawed at the beginning (0 h), followed by CHO DP-12 (mTagBFP) (24 h), CHO DP-12 (mOrange2) (48 h), and CHO DP-12 (Cerulean) after 72 h. Each thawed derivative was initially cultivated for 72 h. Then, shake flask cultures were individually inoculated to 0.3 ⋅ 10^6^ cells ml^− 1^ (40 ml shaking flask, Corning). Each cell line derivative was cultivated for different time periods and harvested from a different growth phase to inoculate the mixed cultures. A schematic schedule is shown in ESM Fig. S3, and the summarized culture age and growth phases are shown in Table 2.

**Table 2:** Cell line derivatives and corresponding culture growth phases

Cell line derivatives	Preculture time	Assigned growth phase
CHO DP-12 (Cerulean)	72 h	Exponential phase (EX)
CHO DP-12 (mOrange2)	96 h	Late exponential phase (LE)
CHO DP-12 (mTagBFP)	120 h	Transition phase (TP)
CHO DP-12 (Venus)	144 h	Stationary phase (SP)

Out of these cultures (Table 2), three mixed aged shake flasks were inoculated to a final cell concentration of 1 ⋅ 10^6^ cells ml^− 1^ (0.25 ⋅ 10^6^ cells ml^− 1^ for each preculture). As a control, mixed exponentially growing cells of each cell line derivative, each taken from precultures that lasted 72 h, were cultivated. Sampling was performed twice a day.

#### Bioreactor cultivation

A time-resolved investigation of the dynamics of the mixed aged cultures (the same as previously mentioned) was investigated in a bioreactor cultivation (Medorex Vario 1000, Medorex, Germany). The working volume at the beginning was 100-ml fresh medium, constituted as explained above (Preculture). pH value was controlled at 7.1 with CO_2_ sparging or the addition of $0.5~\text {mol~l}^{-1}~\text {Na}_{2}\text {CO}_{3}$. Agitation was at 400 rpm and the temperature at 36.8 ^∘^C. Dissolved oxygen was controlled at 40*%* minimum, and pure oxygen was sparged submersely if necessary.

#### Analytics

The cell concentration was measured with the particle counter Z2 (Beckman-Coulter). Viability was determined with the DAPI (4$^{\prime }$,6-diamidino-2-phenylindole) method, as explained in [16]. Concentrations of glucose (*c*_Glc_), glutamine (*c*_Gln_), and lactate (*c*_Lac_) were quantified with the YSI 2900D (Yellow Springs Instruments, USA) biochemistry analyzer and ammonium (*c*_Amm_) with an enzymatic test kit (AK00091, nzytech, Portugal). The antibody titer (*c*_Ab_) was determined with a bio-layer interferometry (Octet RED; Pall ForteBio, USA) with protein A biosensors (Pall ForteBio), according according to the manufacturer’s protocol. In case study I, the concentrations after the cell transfer were calculated.

#### Rate calculation

The cell-specific glucose uptake rate was calculated using the measured glucose concentrations at two consecutive time points (*t*_*i*_, *t*_*i*+ 1_) and the measured viable cell density (*X*_v_) as follows:

2 $$ q_{\text{Glc}}(t_{i},t_{i+1}) = \frac{c_{\text{Glc,i}}-c_{\text{Glc,i}+1}}{t_{i+1}-t_{i}} \cdot \frac{2}{X_{\text{v,i}+1}+X_{\text{v,i}}}. $$

The antibody production rate was calculated with the antibody titer at two consecutive time points:

3 $$ q_{\text{Ab}}(t_{i},t_{i+1}) = \frac{c_{\text{Ab,i}+1}-c_{\text{Ab,i}}}{t_{i+1}-t_{i}} \cdot \frac{2}{X_{\text{v,i}+1}+X_{\text{v,i}}}. $$

## Results and discussion

This study investigated the formation and dynamics of cell population heterogeneities in mammalian cell culture processes using multi-color labeled cell line derivatives. First, the results of the high passage cultivation (case study I) are presented, followed by the investigation of preculture treatment in mixed cultures (case study II).

### High passage cultivation (case study I)

#### 3D distribution due to RGB-marking

**Fig. 1:**
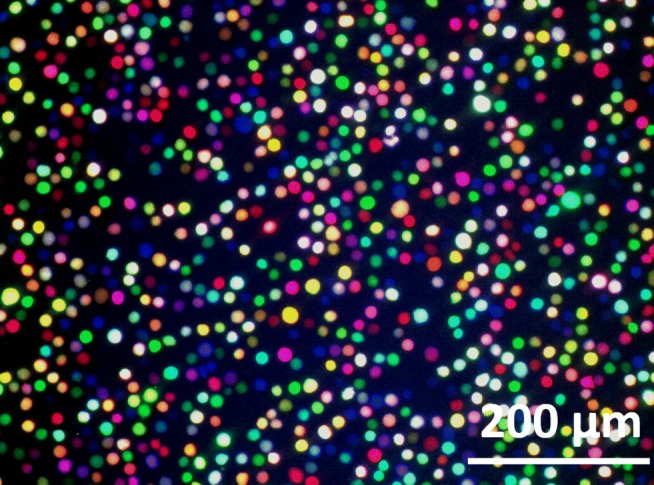
Microscopic image of RGB-marked CHO DP-12 cells at 0 day (case study I); cells were cryo-stored, thawed, and cultivated before imaging (see Preculture); microscope: IX81, camera: color View II, software: CellˆP (all Olympus, Germany), lamp: HBO 103W/2 (Osram, Germany), fluorescence filter: Cerulean, F36-710 (AHF Analysentechnik, Tübingen); Venus, U-MNIBA2 (Olympus); mCherry, U-MWIG2 (Olympus); fluorescence images were edited (GIMP 2.10.12) for white and color balancing; corresponding phase contrast image is shown in ESM Fig. S4 A

The RGB-marked cells express just one or mixtures of the red, green, or blue fluorescent proteins (see Table 1). A microscopic fluorescence image showing the color spectrum of the transduced cells can be seen in Fig. 1 (corresponding phase contrast in ESM Fig. S4 A).

**Fig. 2:**
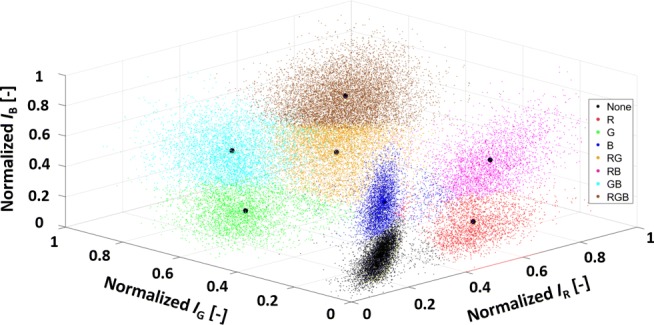
3D plots of RGB-labeled cells during exponential growth phase (case study I), normalized as shown in Flow cytometry, colors were applied to visually distinguish the cell populations, data of shake flask one at *t* = 0 day (High passage cultivation (case study I)). *I*_B_, intensity Cerulean; *I*_G_, intensity Venus; *I*_R_, intensity mCherry

The cells can be assigned to a position in a 3D space based on the flow cytometer measurements, spanned by the normalized fluorescence intensities (Eq. 1). As can be seen in Fig. 2, different cell clusters are formed at the corners of the 3D cube. These clusters are characterized by no, one, or combinations of fluorescence intensity levels.

The cells can be assigned to a non-stained cluster at the lower front (black, (13.8 ± 0.13)*%* (average ± standard deviation), *n* = 3 flasks) and three single-colored populations at the individual lower corners with (8.56 ± 0.21)*%* for the red, (8.66 ± 0.30)*%* for the green, and (7.76 ± 0.09)*%* for the blue population. Double transduced cells are present in the RG cluster with (10.8 ± 0.12)*%*, BR with (7.19 ± 0.04)*%*, and GB with (20.6 ± 0.65)*%*. The triple transduced cells (RGB) account for (22.7 ± 0.84)*%*. In general, each cell is labeled differently and can form individual populations. However, eight cell clusters with different fluorescence intensities were identified to be separated due to the lentiviral transduction. This enables the tracking of changes in the total ratios of the cells in the clusters, including their associated cell numbers. Please notice that different 3D distributions are generally obtained for different MOIs [27, 30, 38].

#### Change in population heterogeneity

**Fig. 3:**
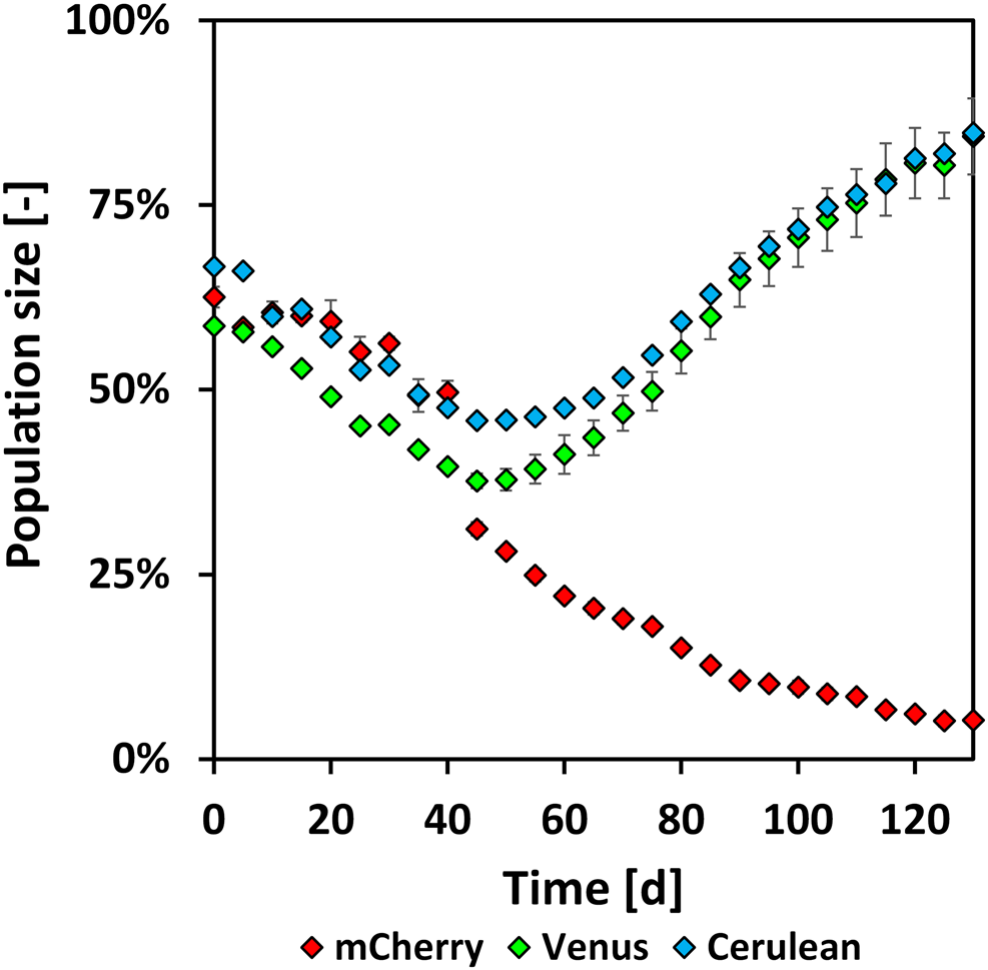
Time-dependent changes of the gated population size (i.e., % of positive gated cells, see Flow cytometry) during high passage cultivation (case study I); symbols (diamonds) average of the three high passage shake flasks cultivated in parallel; error bars show the standard deviation of biological triplicates; exemplary individual histograms are shown in Fig. 4 and ESM Fig. S5 and ESM Fig. S6 (see Flow cytometry for details about flow cytometry)

Three shake flasks were cultivated in parallel for 130 days total, and the cells were passaged every 5 days (see High passage cultivation (case study I)). The fluorescence intensities were quantified, and microscopic images after 110 days are shown in ESM Fig. S4 C and ESM Fig. S4 D. Please notice that the intermediate intensity of Cerulean is not seen due to the high intensity of Venus and that the cells in ESM Fig. S4 D appear to be mostly green. The changes of the gated population sizes over time, as determined by flow cytometry (Flow cytometry), are shown in Fig. 3.

**Fig. 4:**
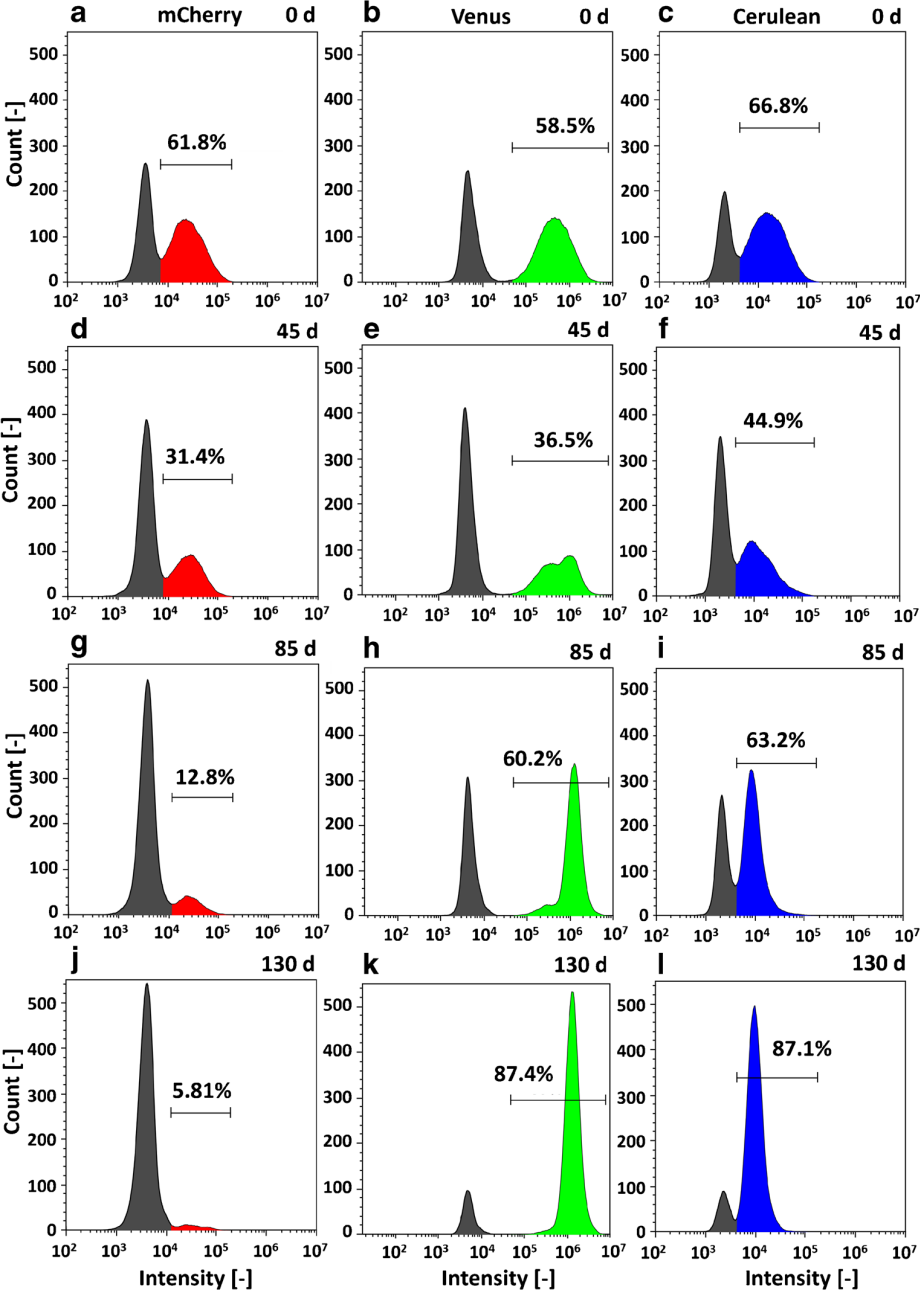
**a**–**l** Change of the RGB fluorescent protein intensities for four different time points (*t* = 0 day,45 days,85 days, and 130 days) and exemplary for culture one (case study I); cultures two and three show the same trend and are shown in ESM Fig. S5 and ESM Fig. S6

To better understand the changes of the fluorescence signals individually, the investigated RGB fluorescent protein intensities at 0 day, 45 days, 85 days, and 130 days are shown in Fig. 4, exemplary for shake flask culture one. In cultures two and three, similar changes were observed, as can be seen in ESM Fig. S5 and ESM Fig. S6. The deviation of the different shake flask cultures is depicted in Fig. 3 as the standard deviation.

##### mCherry

The population size of mCherry-positive cells (Fig. 3) started at (62.5 ± 1.40)*%* and slightly decreased until *t* = 40 days (49.6*%* ± 1.58*%*). Then, the percentage of mCherry strongly decreased until (4.01 ± 0.31)*%* after *t* = 130 days. In the individual intensity plots, mCherry-positive signals (Fig. 4a, d, g, j) started at 61.8% at *t* = 0 day with a large number of cells with different fluorescence intensities, forming a rather wide peak (typical in polyclonal population histograms). Then, the amount of mCherry-positive cells decreased to a negligible amount of 5.81% (*t* = 130 days). At the same time, the amount of mCherry-negative cells (intensities below 1 ⋅ 10^4^) increased accordingly.

##### Venus

At *t* = 0 day, the population size of Venus cells was (58.5 ± 0.46)*%* (Fig. 3) and declined for the first 40 days. After that, the population size of Venus-gated cells increased until *t* = 130 days with (84.3 ± 5.16)*%*. 58.5% of the Venus-positive cells (Fig. 4b, e, h, k) were detected at *t* = 0 day with a comparable shape of the histogram as for mCherry. At *t* = 45 days, the number of Venus-positive cells decreased (36.5%), while at the same time, the number of low signal intensities increased. Interestingly, two peaks can be suspected in the shape of the Venus-positive gate with a lower peak (approx. at 3 ⋅ 10^5^) and one of higher intensity at 1.5 ⋅ 10^6^. At *t* = 85 days, the high-intensity peak rose further, and the percentage of Venus-positive cells increased to 60.2% with an amplitude intensity of 1.5 ⋅ 10^6^, which is comparable to the formerly (*t* = 45 days) identified peak of higher intensity. A small number of cells were still present at the position of the low-intensity peak (*t* = 45 days) at 3 ⋅ 10^5^, but their contribution was negligible. On day 130, the histogram changed, and the percentage of Venus-positive cells was 87.4% with an average signal intensity identical to the previously identified peak of high intensity (1.5 ⋅ 10^6^). The shape of the peak was narrow, and the number of non-positive cells decreased. In summary, a population with narrow fluorescence intensity appeared at *t* = 45 days, became dominant until *t* = 85 days, and almost entirely over-grew the culture until *t* = 130 days.

##### Cerulean

The gated population size of Cerulean accounted for (58.6 ± 0.38)*%*(*t* = 0 day) (Fig. 3) and further increased until (84.8 ± 4.41)*%* at *t* = 130 days, which was comparable with Venus. 66.8% of cells were Cerulean-positive (Fig. 4c, f, i, l) at 0 day with a comparable shape, as explained previously. A second population (*t* = 45 days, at 1 ⋅ 10^4^), comparable with the Venus signals, arose and over-growing of this population at *t* = 85 days, and *t* = 130 days (87.1% Cerulean-positive) was observed with a narrow intensity shape. In comparison with the Venus signals, the second peak was at a lower intensity.

Overall, a shift in the fluorescence intensities was observed with a decrease in the number of mCherry-positive cells and an increase in the number of Venus- or Cerulean-positive cells. The individual signal intensity of Venus increased while the Cerulean intensity decreased, and the histograms alone imply a change of the cell populations during the high passage cultivations.

#### 3D population plots

**Fig. 5:**
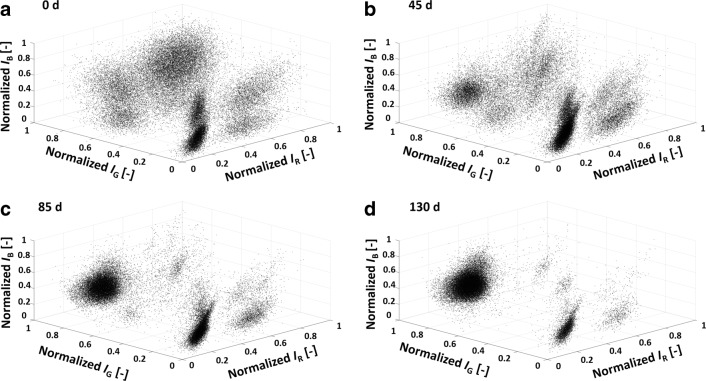
**a**–**d** 3D plots of RGB-marked cells during high passage cultivations (case study I); exemplary at 0 day, 45 days, 85 days, and 130 days for shake flask culture one (see High passage cultivation (case study I)), *I*_B_, intensity Cerulean; *I*_G_, intensity Venus; *I*_R_, intensity mCherry

A 3D representation of the fluorescence intensities is necessary to evaluate the number and shape of the cell population heterogeneities in detail (e.g., check for multiple or single populations) since these cannot be depicted from the intensity histograms alone (Figs. 3 and 4, respectively). As can be seen in Fig. 5a, all 8 populations (see 3D distribution due to RGB-marking) were present at 0 day with the formerly introduced distribution.

At 45 days (Fig. 5b), a cell population with a high Venus and intermediate Cerulean intensity arose at *I*_R_ ≈ 0.2,*I*_G_ ≈ 0.7, and *I*_B_ ≈ 0.4. At *t* = 85 days, the number of cells in this population increased (Fig. 5c), while all other populations are reduced in size. The said population further increased in size, clearly overgrowing all other labeled populations at *t* = 130 days (Fig. 5d). The spherical form of the population is an indicator of its uniformity [38]. The quantity of the non-labeled cell population at the front corner is comparable with 0 day, while no other population is present in a relevant amount.

#### Cell growth, metabolism, and productivity

**Fig. 6:**
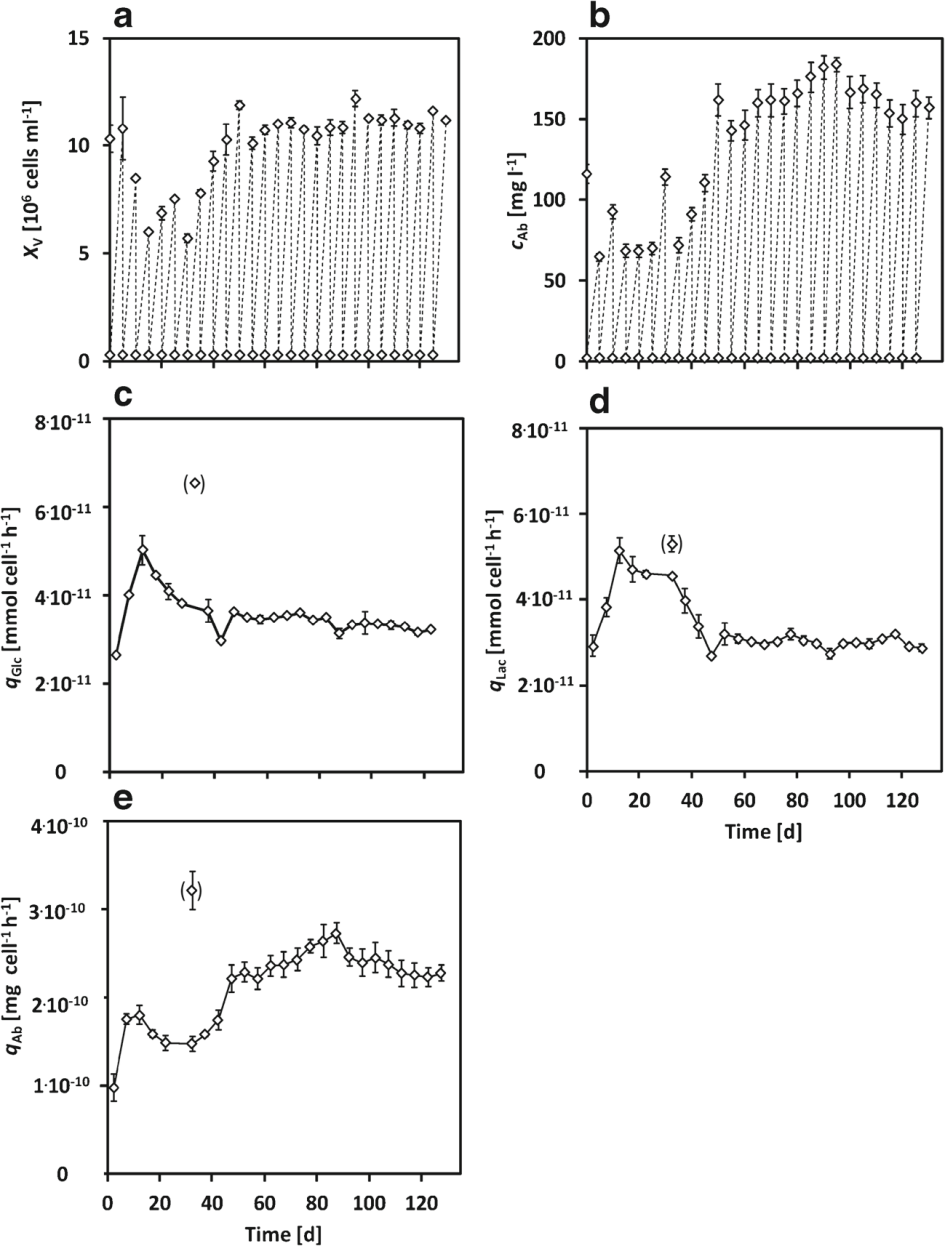
**a**–**e** Mean experimental results (case study I, diamonds) of three parallel high passage shake flask cultivations (see High passage cultivation (case study I)); error bars show the standard deviation of biological triplicates (each measured three times); cells were transferred every 5 days rates in brackets were excluded

The average concentrations and calculated rates are shown in Fig. 6.

##### Cell growth

For the first 40 days, fluctuations occurred in the viable cell density (Fig. 6a) in the range of $X_{\mathrm {v}}=(8.63~\pm ~2.03)~\cdot ~10^{6}~\text {cells~ml}^{-1}$ with a decline in *X*_v_ during the first 15 days and a rise between *t* = 30 − 50 days with a nearly constant $X_{\mathrm {v}} = (11~\pm ~0.50)~\cdot ~10^{6}~\text {cells~ml}^{-1}$ after 45 days.

##### Glucose and lactate

Glucose (ESM Fig. S7) was constantly consumed during high passage cultivations and was not below $c_{\text {Glc}}\approx ~20~\text {mmol~l}^{-1}$ at the end of each culture period (5 days). However, less glucose was consumed at the beginning of the high passage cultivations until 50 days. The cell-specific glucose uptake rate (Fig. 6c) shows a declining trend with a maximum of *q*_Glc_ = (5.00 ± 0.19) ⋅ 10^− 11^ mmol cell^− 1^ h^− 1^. After that, it increased and was stable for the rest of the high passage cultivation. The lactate concentration was constant at approx. $c_{\text {Lac}} = (20.4~\pm ~0.51)~\text {mmol~l}^{-1}$, without huge variations. Such a maximal lactate concentration was also previously observed for shake flask cultivations of CHO DP-12 cells [39], presumably due to the uncontrolled pH value. The cell-specific lactate production rate (Fig. 6d) increased first and decreased contrarily to the glucose uptake rate and was also constant after 50 days.

##### Antibody

The antibody (Fig. 6b) was produced in comparably low concentrations for the first 40 days with $c_{\text {Ab}} = (84~\pm ~21)~\text {mg~l}^{-1}$. After this, the titer increased up to $c_{\text {Ab}} = (184~ \pm ~4)~\text {mg~l}^{-1}$ until *t* = 95 days, which corresponds to an increase of 220%. Interestingly, the antibody concentration further decreased until the end of the high passage cultivations even though *X*_v_, *c*_Glc_, and *c*_Lac_ remained constant. The same trend was observable in the cell-specific antibody productivity (Fig. 6e), which increased after 37.5 days to a maximum of $q_{\text {Ab}} = (2.7~\pm ~0.1)~\cdot ~10^{-10}~\text {mg~cell}^{-1}~\text {h}^{-1}$ (*t* = 92.5 days). Then, it constantly declined until $q_{\text {Ab}} = (2.3~\pm ~0.1)~\cdot ~10^{-10}~\text {mg~cell}^{-1}~\text {h}^{-1}$ (*t* = 127.5 days), which corresponds to a reduction in productivity of − 15%.

#### Discussion

Cell populations with low production rates have presumably higher proliferation rates and would, in theory, overgrow the culture [40, 41]. In this study, a sub-population with a higher growth rate, productivity, and unique RGB-based color (high green, intermediate blue, see ESM Fig. S4 D) developed in the cultures. This population overgrew the other cell populations and a change in the fluorescence intensities (Figs. 3 and 4, respectively) and cell populations (Fig. 5) were observed. Brenière-Letuffe et al. [38] found that the sub-clonal diversity in human-induced pluripotent stem cells was reduced to less than ten clones after 38 passages. Here, the rise of the same cell population was observed in all three parallel high passage cultivations and therefore led to the conclusion that it was already present in the preculture and was not caused by mutation during the monitored culture period.

Beckmann et al. [33] investigated long-term cultivations of CHO DP-12 cells (passaged every 3 days). They found that the cell density at the end of each passage rose over time and that the antibody titer increased during the first 95 days and decreased until no production was measured after 420 days. By the same time, the glycosylation and expression of anti-stress proteins increased [33]. In this study, cell growth and productivity increased for the first 45 days and then remained constant, even if the cells were passaged every 5 days (viability above 95%). By the same time, the antibody titer and cell-specific productivity (Fig. 6) first increased until approx. *t* = 100 days and then started to decline. These findings are supported by [33, 42, 43], who observed that reduced antibody production occurred during long-term cultivation, which was attributed to the loss of gene copies responsible for the expression of the antibodies. Alternatively, a single or multiple sub-populations or clones might have formed out of the predominant population after *t* = 100 days, which still expresses the same fluorescent proteins and cannot be distinguished by flow cytometry. The transduction with LeGO vectors (RGB-marking) labels the cells at the time of transduction and timely changes are generally in comparison with this time point. Therefore, further population changes within the observed unique sub-population cannot be detected and would require renewed RGB transduction [27]. Moreover, population-independent whole culture changes in expression and cell growth might have occurred (e.g., random loss of genes for expression), which cannot be distinguished from the individual formation of sub-populations [33, 42]. No changes were observed for glucose and lactate metabolism, which were not affected by the change in productivity and growth. The genetic integration of RGB genes was shown to be stable [27]. As a general caveat, the lentiviral transduction itself could lead to sub-population changes (insertional mutagenesis), which was not relevant in our studies due to their low probability [44] and the low number of transduced cells (Generation of labeled cell line derivatives).

Overall, the results provide an insight into the highly dynamic formation of population heterogeneity with potential sub-populations and clonal outgrowth and their impact on the cell culture process. Based on our findings, long-term maintenance cultures and exhausting seed-trains (i.e., inoculation train) should be avoided in view of process stability and cell population integrity.

### Mixed cultures (case study II)

Case study II investigated the impact of preculture treatment on mixed cultures, using stably transduced cell line derivatives expressing different fluorescent proteins (Cerulean, mOrange2, mTagBFP, Venus, respectively, see Mixed cultures (case study II)). Therefore, precultures were grown up to different growth phases and then pooled to inoculate mixed shake flask cultures and a bioreactor culture with AFC measurements in parallel. Mixed exponentially grown shake flask cultures were cultivated as negative control.

#### Analysis of cell populations

**Fig. 7:**
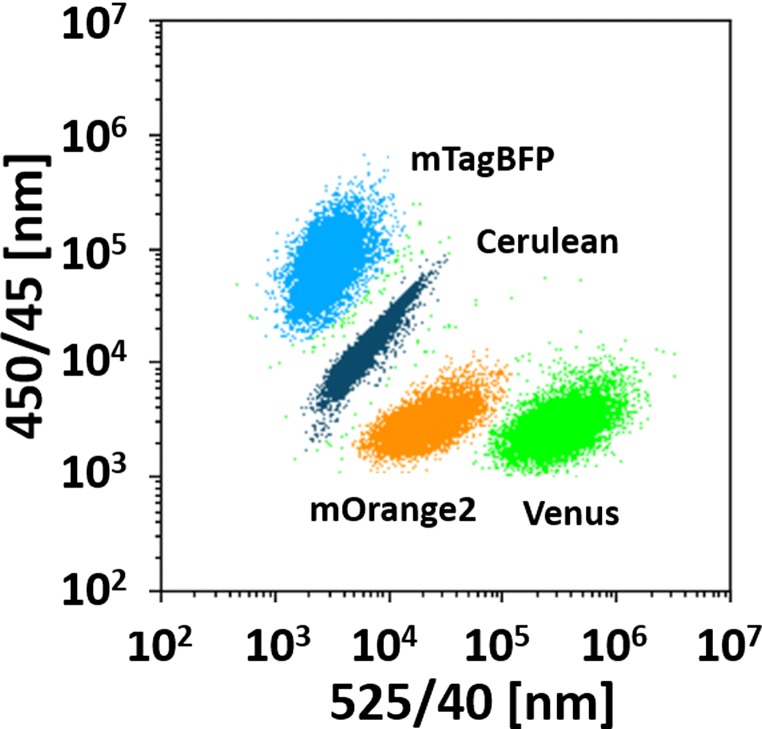
Dot plot of mixed cultures consisting of four differently labeled cell line derivatives (case study II, Mixed cultures (case study II)) as analyzed by flow cytometry; microscopic images in ESM Fig. S8, axes are the intensities in the used filters in flow cytometry

The different fluorescent proteins (Mixed cultures (case study II)) for the cell line derivatives were chosen to separate the populations by flow cytometry. Here, only two lasers (405 nm and 488 nm, respectively) and two filters (450/45 nm and 585/42 nm, respectively) were needed to clearly distinguish all four populations, which are shown in Fig. 7. Microscopic images of the individual cell populations expressing different fluorescent proteins are shown in ESM Fig. S8.

No compensation or sophisticated gating strategies were necessary, allowing a fast and reproducible analysis of mixed populations and their corresponding sub-populations. Furthermore, the dynamics of the population changes can be evaluated if samples, acquired at different time points, are compared.

#### Mixed shake flask cultures: population heterogeneity

**Fig. 8:**
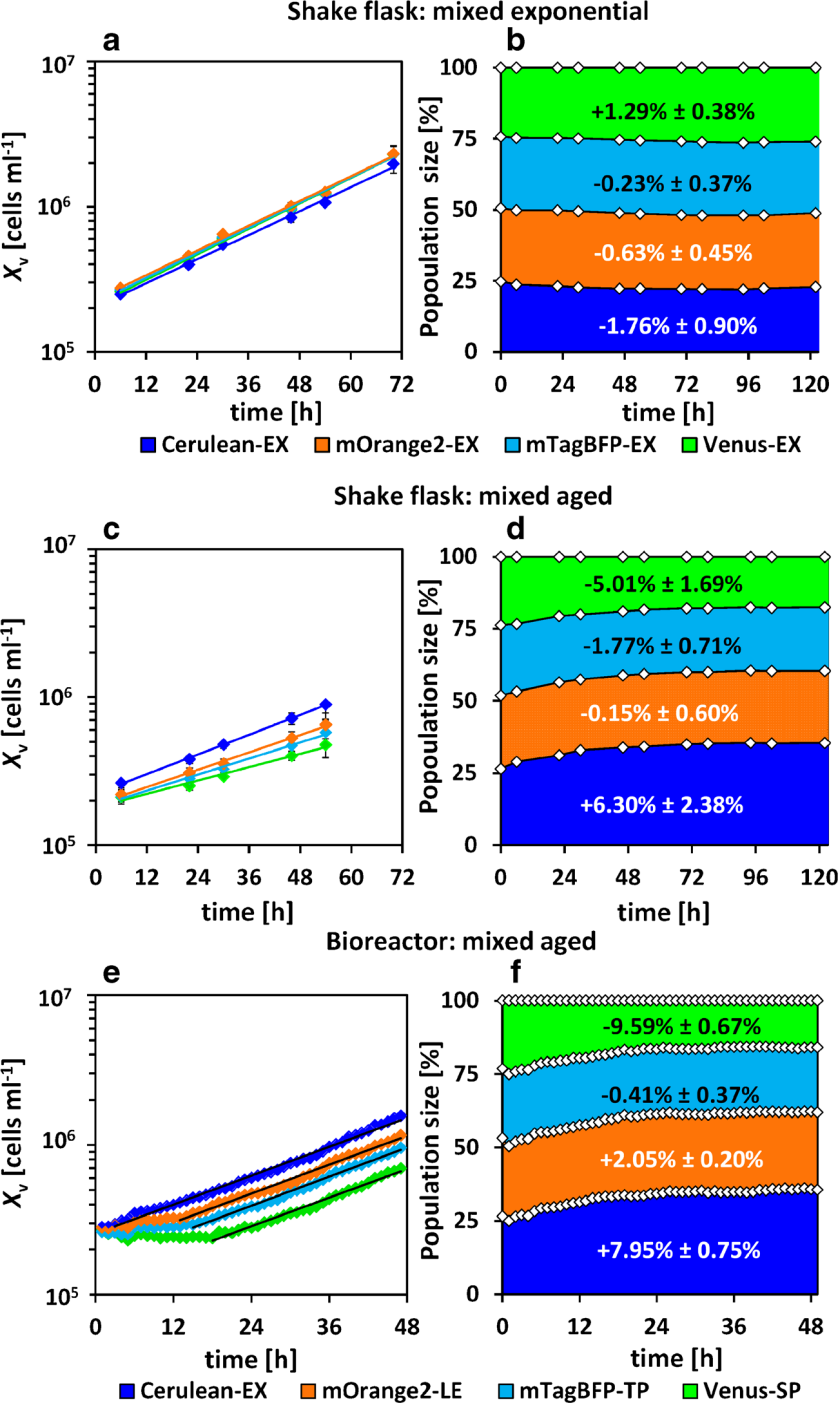
Average experimental results (case study II, diamonds) of the mixed exponential cultures (**a**, **b**, shake flask, *n* = 3), mixed aged cultures (**c**, **d**, shake flask, *n* = 3), mixed aged bioreactor culture (**e**, **f**, *n* = 1); details of individual populations are shown in Table 2, error bars represent standard deviation of biological triplicates (Mixed cultures (case study II))

The logarithmic plots of the viable cell density related to the individual cell populations and the percentage of the individual populations in the mixed cultures are shown in Fig. 8a–d for the shake flasks and Fig. 8e and f for the bioreactor experiment (AFC). The lines shown represent the individual slopes of the logarithmically plotted experimental data, which are proportional to *μ*.

##### Shake flask cultures

For the mixed exponentially growing cells, the population-dependent growth (Fig. 8a) is narrow, which indicates a comparable cell growth for all four sub-populations. The percentage of cells in the individual populations (Fig. 8b) changed only very little and was stable for the mixed exponential cultivations. The corresponding lines for the cell population–dependent growth (Fig. 8c) were widely distributed. The slopes for the different populations were different, indicating a reduced *μ* for mOrange2-LE, mTagBFP-TP, and Venus-SP compared with the exponentially growing populations.

The population size changed during the first 48 h (Fig. 8d) with an increase of ((6.30 ± 2.38)%, first measurements vs. last measurements, total population) in the Cerulean-EX population, which equals an increase of approx. 24% in the individual population (% end divided by % beginning). Only low relative changes were present for mOrange2-LE cells, but the mTagBFP-TP population decreased by (1.77 ± 0.71)% (total population, ≈ 7*%* individual). The percentage of Venus-SP cells decreased by about (5.01 ± 1.69)% (total population), and the individual population decreased by approx. − 20%. These changes occurred during the first 48 h of cultivation, and the distribution of the populations was constant thereafter.

##### Bioreactor cultivation with automated flow cytometry

The growth effects at the beginning of the mixed aged cultures were monitored in more detail using automated flow cytometry in a bioreactor experiment (Flow cytometry). Therefore, samples were automatically drawn every hour for 48 h cultivation time. Different lag phases were observed in the mixed culture, as can be seen in (Fig. 8e). Note that such observation was only possible due to high sampling frequency at the beginning of cultivation. The cell density of the Cerulean-EX culture had only a low lag phase of 3 h, whereas mOrange2-LE and mTagBFP-TP had 13 h and 15 h, respectively. The Venus-SP population starts to grow exponentially after 18 h. Interestingly, the slope (*μ*) of the adapted exponential growth data was comparable for all cultures (*μ* = 0.037 ± 0.05 h^− 1^) when they entered the exponential growth phase, which was determined and visualized through the parallel lines (Fig. 8e). The population-dependent change of the composition was high (Fig. 8f) and the Cerulean-EX population grew faster than the other three populations with an increase in the population size of (7.95 ± 0.75)% (first three measurements vs. last three measurements, total population) and approx. + 33% based on the individual cell population (first three measurements vs. last three measurements). Only a small relative increase was measured for the mOrange2-LE population ((2.05 ± 0.20)*%*), and the mTagBFP-TP population decreased by (0.41 ± 0.37)% (total). The Venus-SP population decreased (− 9.59 ± 0.67)% (total population) and approx. − 26% based on the individual change. For all populations, the proportions of the individual populations were stable after approx. 24 h.

#### Bulk growth and productivity

**Fig. 9:**
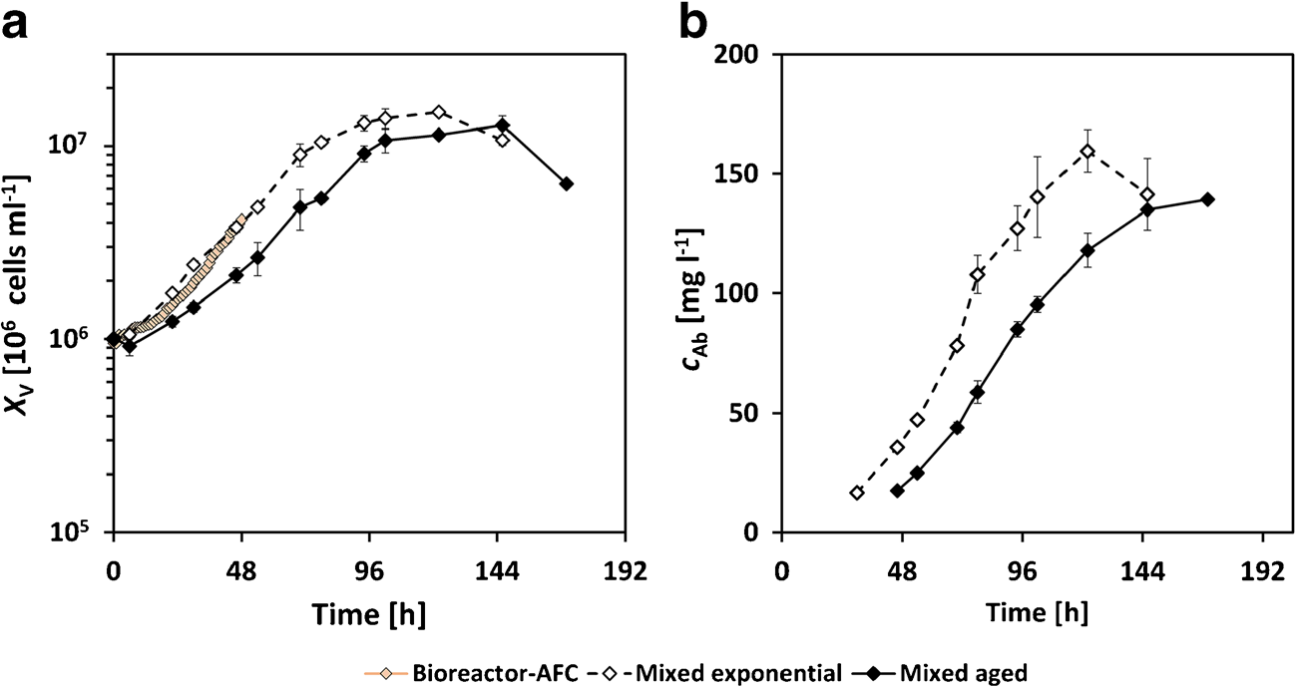
**a**, **b** Experimental results (Mixed cultures (case study II), diamonds) of the mixed exponential cultures (dashed line), the mixed aged cultures (solid) and the bioreactor cultivation with automated flow cytometry (Flow cytometry) and mixed aged cells; error bars represent standard deviation of biological triplicates

The bulk changes in the viable cell density and antibody titer are shown in Fig. 9.

For the mixed exponential cultures (shake flasks), the viable cell density (Fig. 9a) increased without any observable lag phase from 1 ⋅ 10^6^ cells ml^− 1^ until a maximum concentration of (15.0 ± 0.57) ⋅ 10^6^ cells ml^− 1^ at 122 h. At the same time, the antibody titer (Fig. 9b) increased up to $c_{\text {Ab}}=(159.5 \pm 4.4)~\text {mg~l}^{-1}$ (122 h). Both measurements were comparable with earlier studies with the parental cell line and show the expected growth and antibody formation behavior [39]. For the mixed culture with the differently aged populations (shake flasks), a lower cell growth with a lag phase during the first 48 h was observed. After that, cell growth was comparable with the mixed exponential cultures. However, the maximal cell number was reduced ($X_{\mathrm {v}}=(12.81 \pm 1.50)~\cdot ~10^{6}~\text {cells~ml}^{-1}$ at 146 h) and achieved later than in the exponentially grown cultures. Comparable trends with higher substrate uptake and earlier metabolite formation were present in the antibody titer (Fig. 9b) and the concentrations of glucose, glutamine, lactate, and ammonium, which are shown in ESM Fig. S9. The bioreactor experiment with AFC (Fig. 9a) showed a reduced growth at the beginning and was then between the mixed exponential and mixed aged data. Overall, a different bulk behavior was observed in the mixed exponential cultures compared with the mixed aged cultures.

#### Discussion

Different growth phases are characterized generally by different bulk growth behaviors and varying metabolic fluxes and regulations [45–48]. The transition of the cultures into the stationary growth phase occurs along with apoptosis and necrosis [49, 50]. Furthermore, growth inhibitory media components can accumulate (e.g., ammonium and putative autocrine factors), which can lead to changes in the metabolism [16, 17, 51]. In this study, a different bulk cell growth was observed for mixed shake flask cultures comprised of populations from different growth phases. The overall cell growth and productivity were reduced compared with mixed exponential shake flask cultures. The analysis of the population-dependent cell growth revealed that the transition and stationary phase–derived populations (mTagBFP-TP and Venus-SP, respectively) started proliferation after a lag phase and did not grow directly after inoculation. This leads to a change in the cell population composition during the first 48 h with absolute population shifts of (13.2 ± 5.38)% (sum of all changes, mixed aged shake flask cultures), which were not present during the mixed exponential shake flask cultures. It should be noticed that cell growth restored for all populations, even for those cells that had previously entered the stationary phase. Higher shifts in the population composition were observed in the online AFC bioreactor experiment with total changes of (20.1 ± 1.99)% (sum of all changes) and the lag phases correlated to the preculture growth phases. Such variations in the lag time and population changes between the shake flask and bioreactor cultivations can be based on general differences in pH regulation and power input [52, 53].

The identified population changes can affect process variability with different productivities in large scale, e.g., if cells are pooled from different bioreactors for inoculation [6, 22]. Moreover, this study demonstrates the strong impact of preculture treatment on the bulk process performance and the need for an efficient seed train design [54] and scale-up strategy [55]. Regarding the application of the cell labeling concept, the changes in the bulk composition behavior would be not observable if the different populations were not individually marked. In our opinion, there is no better way to compare different cell populations than to grow them in one and the same culture under almost perfectly identical conditions for all populations over the whole experiment. Their only difference would be the color of their fluorescent marker, which is even interchangeable. Thus, the proposed concept is appropriate to understand the dynamics and interactions of cell population heterogeneity in CHO cell cultures.

## Conclusion

This study investigated cell population dynamics in mammalian cell culture processes in two different case studies: In case study I, the cells were lentivirally marked with combinations of three colors, and changes in the productivity and cell growth were identified in high passage cultivations. These shifts were associated with changes in cell populations with just one dominating, potentially monoclonal, population after 130 days. In case study II, population dynamics were assessed in mixed cultures originating from differently labeled populations, each representing a different growth phase. The bulk differences in cell growth were related to changes in the population size. In summary, this contribution provides a novel approach to understanding cell population heterogeneities and their dynamics at the cell population level. This approach is advantageous in the field of bioprocess engineering because different cells and cell populations can be investigated in a non-destructive way and living cells can be separated (e.g., fluorescence-activated cell sorting), which enables a more detailed investigation of the populations (e.g., genotype studies) [38]. Moreover, fluorescence-based analytics (e.g., flow cytometry) are fast and rather simple compared with, e.g., genetic barcoding approaches relying on high-throughput sequencing [25, 28]. Further applications can involve the investigation of population dynamics in continuous cultures and the assessment of different preculture treatments and their interactions.

**Nomenclature**

**Table:** 

Variable	Explanation	Unit
c_i_	concentration of component i	[mmol l^− 1^]
i	Glc, Gln, Lac, Amm, Ab	[-]
*I*_lower_	lower fluorescence boundary	[-]
*I*_normalized_	normalized fluorescence intensity	[-]
*I*_measured_	measured fluorescence intensity	[-]
*I*_upper_	upper fluorescence boundary	[-]
*X*_v_	viable cell density	[cells l^− 1^]
*q*_Glc_	cell-specific glucose uptake rate	[mmol cell^− 1^ h^− 1^]
*q*_Lac_	cell-specific lactate formation rate	[mmol cell^− 1^ h^− 1^]
*q*_Ab_	cell-specific antibody formation rate	[mmol cell^− 1^ h^− 1^]
*μ*	specific growth rate	[h^− 1^]

**Abbreviations**

**Table:** 

Abbreviation	Explanation
Ab	antibody
AFC	automated flow cytometry
Amm	ammonium
B	blue
CHO	Chinese hamster ovary
DAPI	4$^{\prime }$,6-diamidino-2-phenylindole
EX	exponential phase
G	green
LE	late exponential phase
LeGO	lentiviral gene ontology
FSC	forward scatter
Gln	glutamine
Glc	glucose
Lac	lactate
R	red
SP	stationary phase
SSC	side scatter
TP	transition phase

## Electronic supplementary material

Below is the link to the electronic supplementary material.

(PDF 25.1 MB)
