# Supplementary material for: Quantification of the dynamics of population heterogeneities in CHO cultures with stably integrated fluorescent markers
Source: Anal Bioanal Chem. 2020 Mar 4;412(9):2065–80. doi: 10.1007/s00216-020-02401-5 (PMC7072063; doi:10.1007/s00216-020-02401-5)
Supplement: Supplementary file 1 — (PDF 25.1 MB) [file 216_2020_2401_MOESM1_ESM.pdf]

Analytical and Bioanalytical Chemistry

Electronic Supplementary Material

Quantification of the dynamics of population heterogeneities in CHO cultures with stably integrated fluorescent markers

Johannes Möller, Marcel Rosenberg, Kristoffer Riecken, Ralf Pörtner, An-Ping Zeng, Uwe Jandt

1 Automated and online flow cytometry

An automated and online flow cytometry set-up was designed as follows. A needle (diameter: 1 mm, Medorex, Germany) was connected to the bioreactor representing an inline sampling port and a small tubing (diameter: 0.5 mm, length: 60 cm) was connected to the flow cytometer (Cytoflex, Beckmann Coulter, Germany) instead of the sample needle. Sample shaking was turned off and backflushing after measurement was set to a minimum of 1 s to avoid contamination of the bioreactor. This set-up could be used to measure the fluorescence signals online and the samples were neither filtered nor stained prior to measurement. Furthermore, automated measurements with distinct time intervals or at distinct times were performed using the desktop automation software "perfect automation" (Version 2.7.1, available at http://www.perfectautomation.com/). Then, a flow of 60 $\frac{\mu l}{\min}$ was set for 6 min to flush the tube with fresh cultivation broth. The term online was defined according to Biechele et al. (2015) because the sampling time is low (approx. 10 min total) compared to the process dynamics (approx. 22 h for cell cycle) (Biechele et al., 2015). 50000 events were recorded at an appropriate flow rate and the system was turned to standby afterward (Möller et al., 2019).

2 Supplementary figures

*Correspondence to: Möller, Johannes; Hamburg University of Technology, Bioprocess and Biosystems Engineering, Denickestr. 15, K-1516, 21073 Hamburg, Germany, +49 (0) 40 42878 - 3950, e-mail: johannes.moeller@tuhh.de,

†Jandt, Uwe; Hamburg University of Technology, Bioprocess and Biosystems Engineering, Denickestr. 15, K-1516, 21073 Hamburg, Germany, +49 (0) 40 42878 - 2847, e-mail: uwe.jandt@tuhh.de,

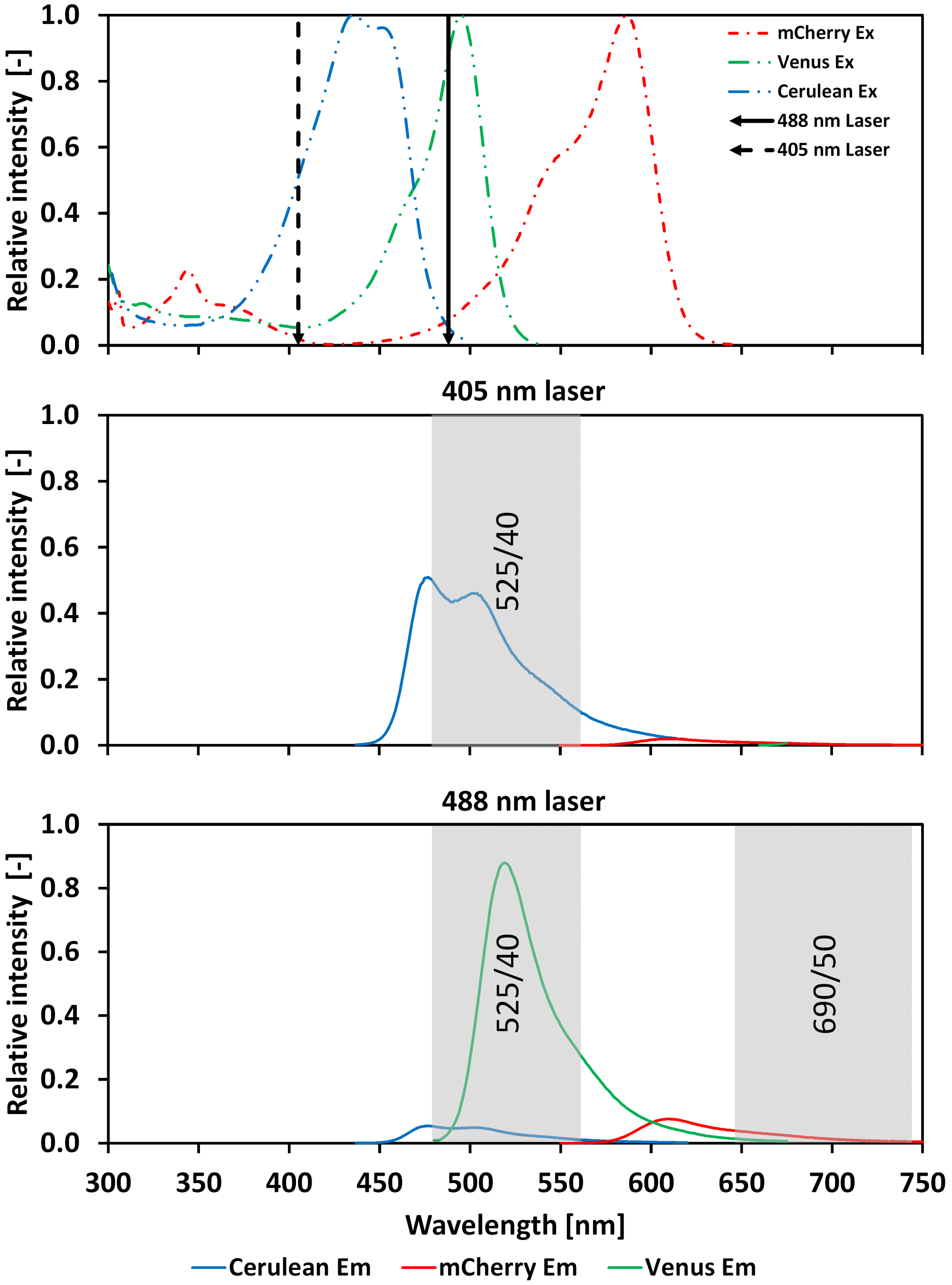

Fig. S1 Excitation and emission spectra of fluorescent proteins lentivirally expressed in CHO DP-12 (Case study I, RGB marking), relative intensities are based on the used lasers and filters (see 2.2.2 in main manuscript); fluorescence spectra were derived from: mCherry (Shaner et al., 2004), Venus (Nagai et al., 2002), Cerulean (Rizzo et al., 2004); all spectra exported from https://www.fpbase.org/ (Accessed: 10.08.2019)

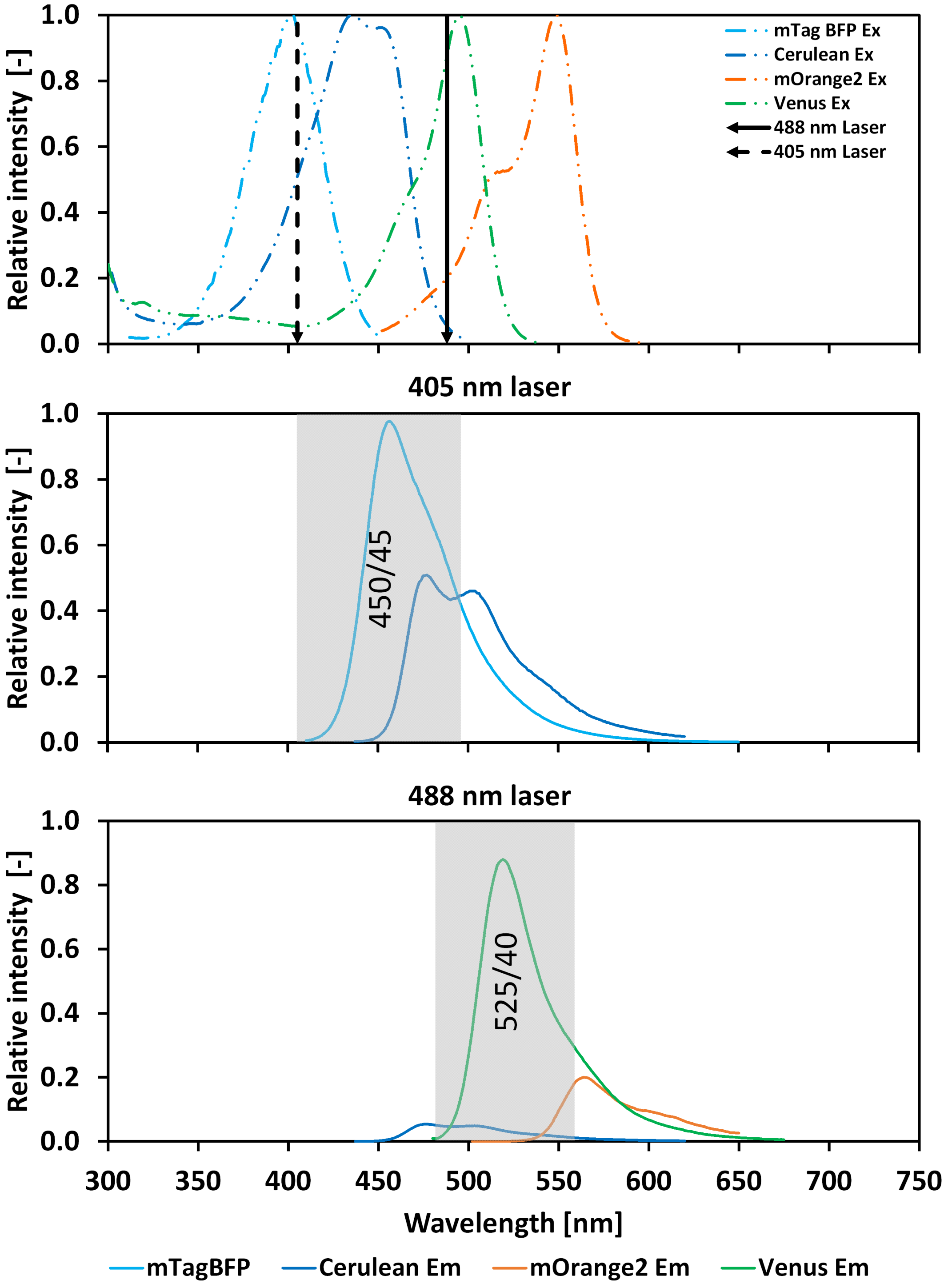

Fig. S2 Excitation and emission spectra of fluorescent proteins used to label single color CHO DP-12 cell line derivatives (Case study II), relative intensities are based on the used lasers and filters (see 2.2.2 in main manuscript); fluorescence spectra were derived from: mTagBFP (Subach et al., 2011), Cerulean (Rizzo et al., 2004), mOrange2 (Shaner et al., 2008), Venus (Nagai et al., 2002), all spectra exported from https://www.fpbase.org/ (Accessed: 10.08.2019)

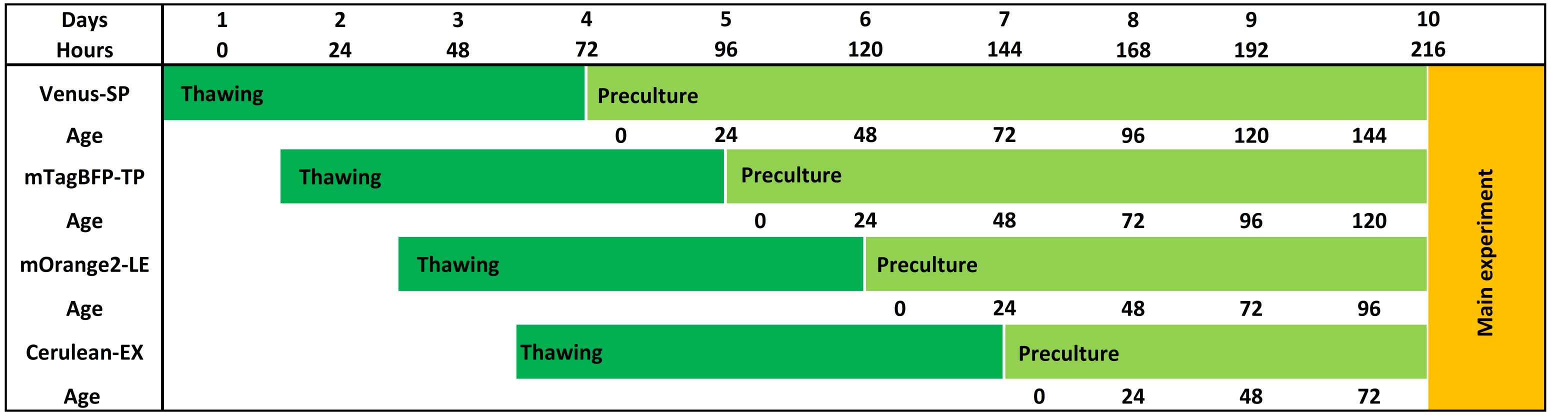

Fig. S3 Time schedule for mixed cultures (Case study II)

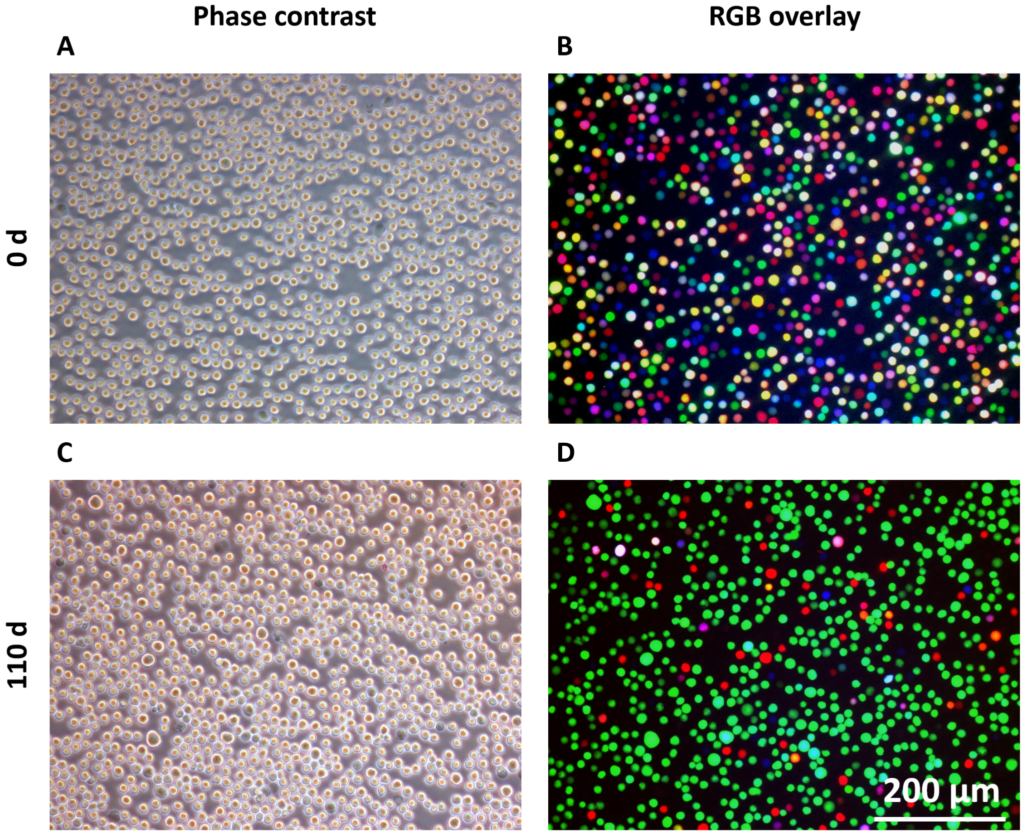

Fig. S4 Microscopic images of RGB-marked CHO DP-12 cells (Case study I), phase contrast (A, C) and fluorescence overlays (B, D) at 0 d and 110 d (used in case study I); cells were cryo stored (2.2.1 in manuscript), thawed and cultivated before imaging (2.1.1 in manuscript); microscope: IX81, camera: Color View II, software: Cellˆ P (all Olympus, Germany), lamp: HBO 103W/2 (Osram, Germany), fluorescence filter: Cerulean - F36-710 (AHF Analysentechnik, Tübingen), Venus - U-MNIBA2 (Olympus), mCherry - U-MWIG2 (Olympus); fluorescence images were edited (GIMP 2.10.12) for white and color balancing, due to the high intensity of Venus, the intermediate high intensity of Cerulean is not seen

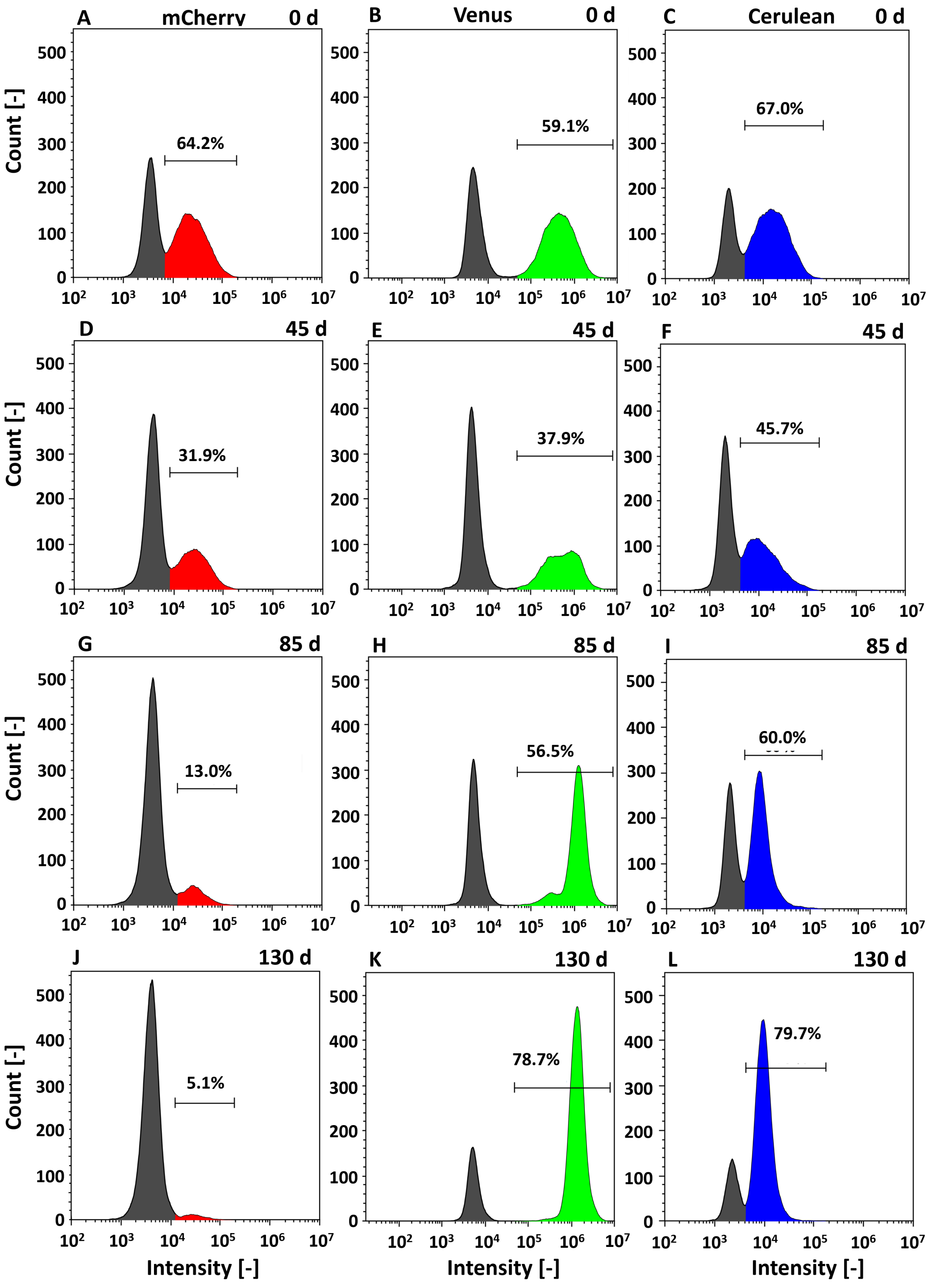

Fig. S5 Timely changes of the RGB fluorescent protein intensities for culture two at t = 0 d, 45 d, 85 d, and 130 d (Case study I)

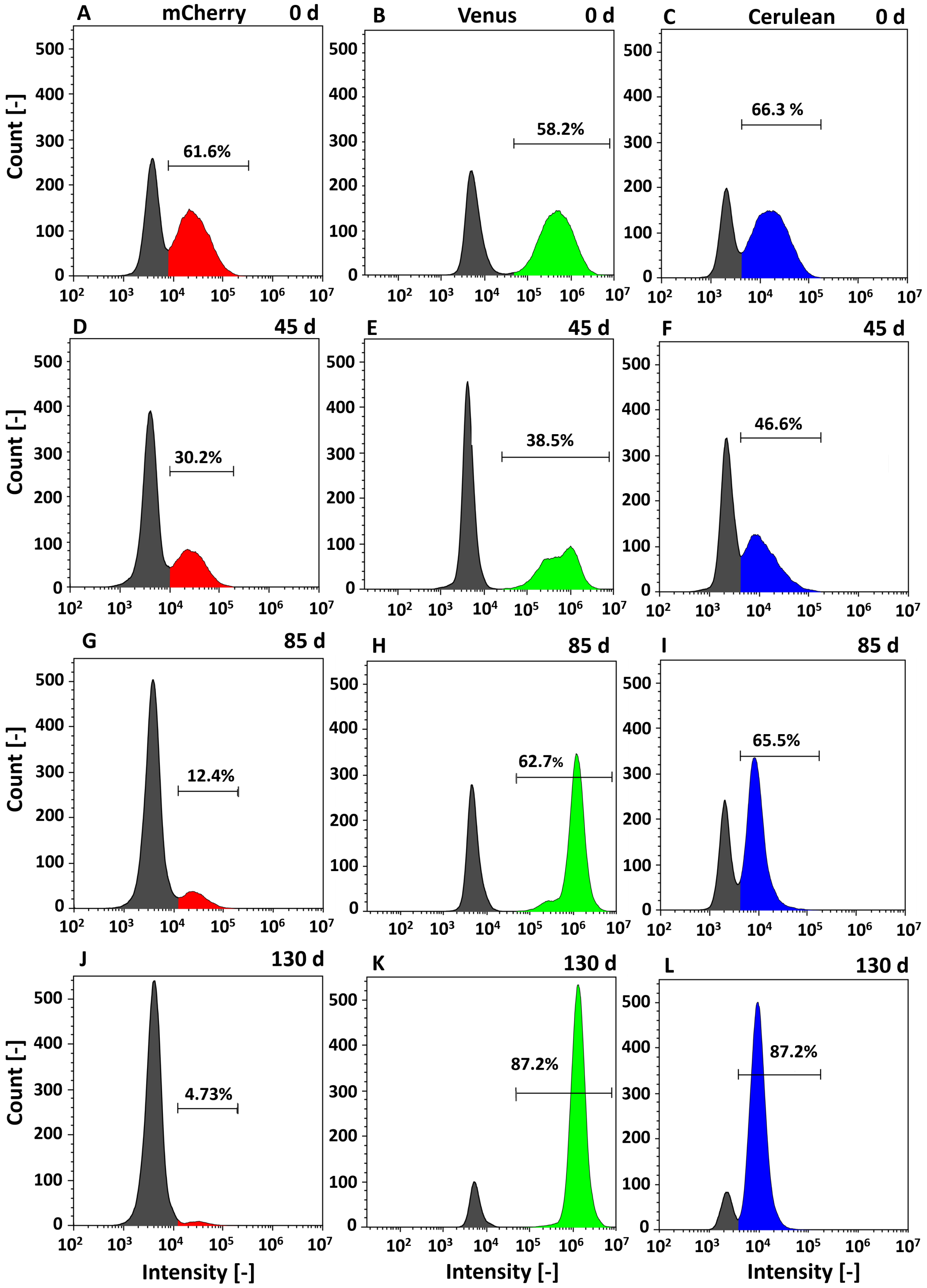

Fig. S6 Timely changes of the RGB fluorescent protein intensities for culture three at t = 0 d, 45 d, 85 d, and 130 d (Case study I)

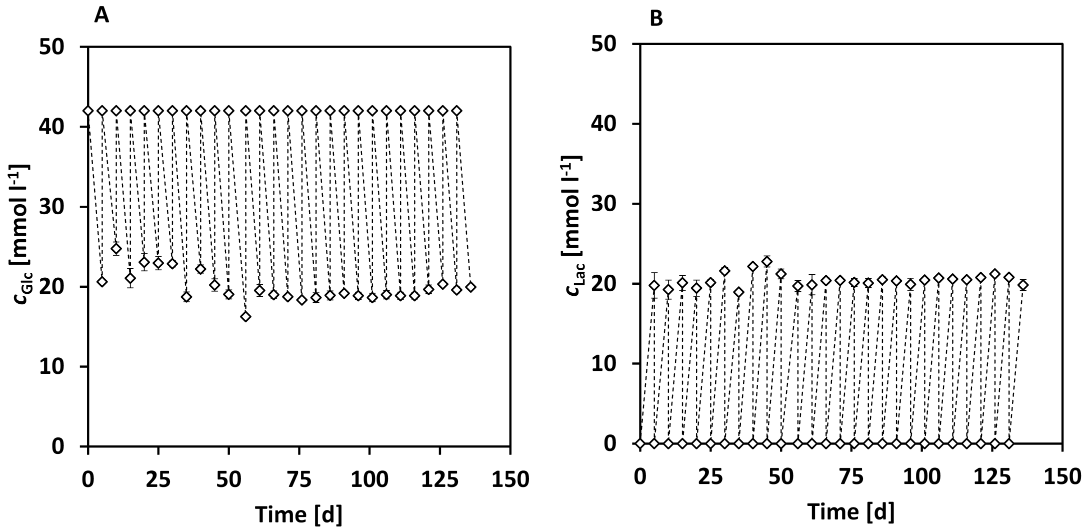

Fig. S7 Mean experimental results (diamonds) of three parallel high passage shake flask cultivations (Case study I); error bars show the standard deviation of biological triplicates (each measured three times); cells were transferred every 5 d

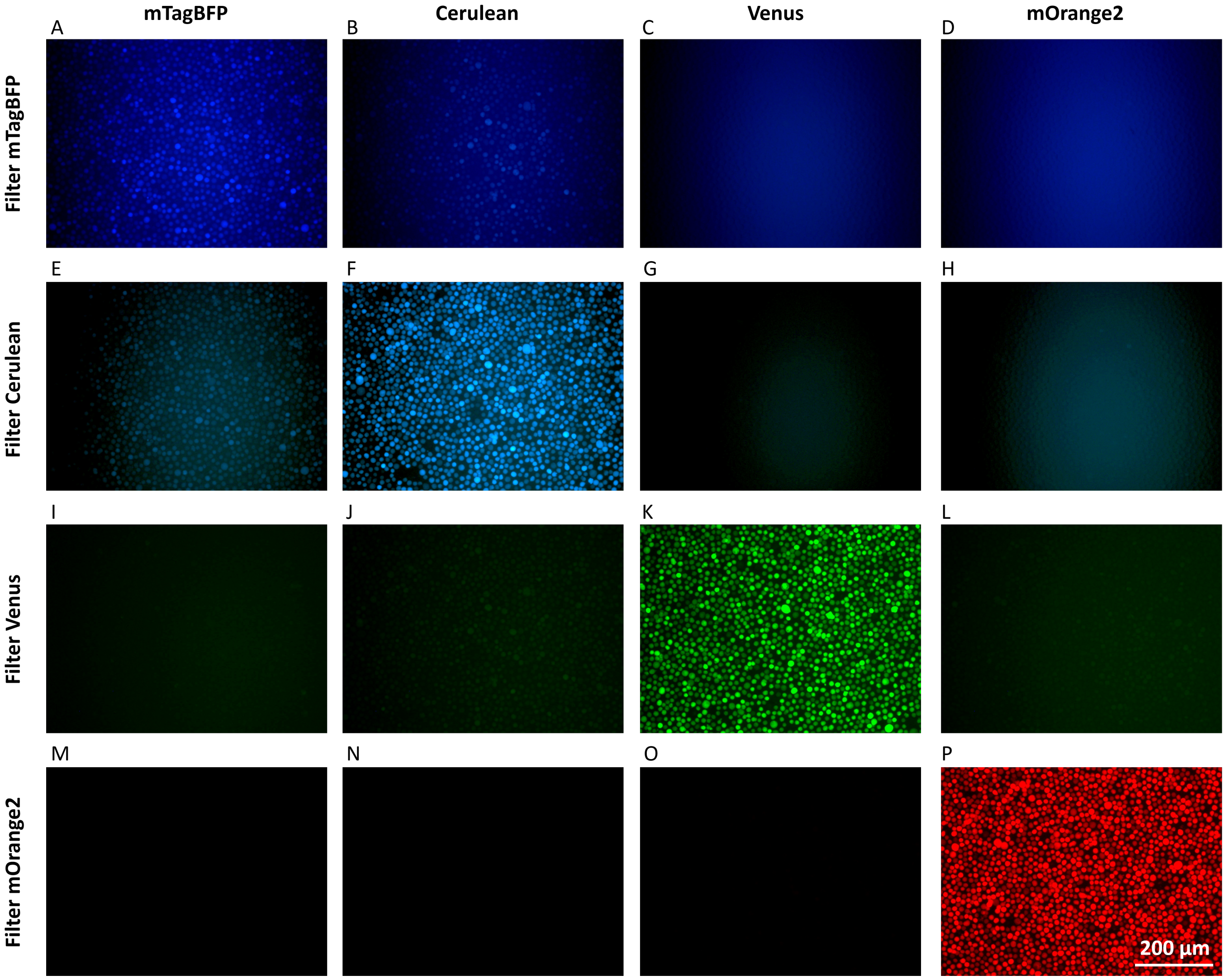

Fig. S8 Microscopic images of four individually labeled derivatives of exponentially grown CHO DP-12 cells (used in Case study II); Each column represents an individual culture recorded in four fluorescent channels; microscope: IX81, camera: Color View II, software: Cellˆ P (all Olympus, Germany), lamp: HBO 103W/2 (Osram, Germany), fluorescence filters: mTagBFP - F41-031 (AHF), Cerulean - F36-710 (AHF), Venus - U-MNIBA2 (Olympus), mOrange2 - U-MWIG2 (Olympus); fluorescence images were edited for each filter (GIMP 2.10.12) for white and color balancing; please note that mTagBFP intensity (A) is rather low in the available filter, but fluorescence identity was proven using flow cytometry, as shown in Figure 7 in main manuscript

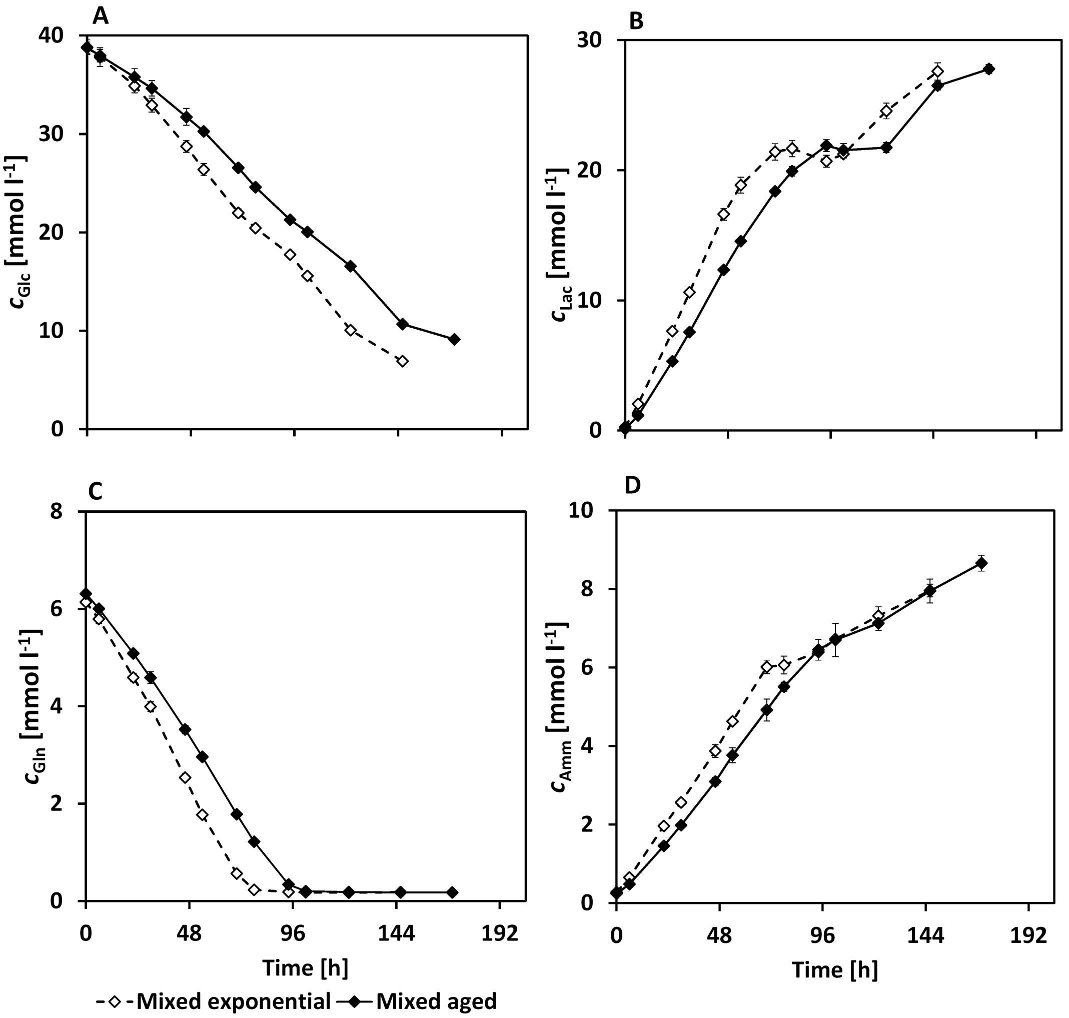

Fig. S9 Average experimental results (Case study II, diamonds) of the mixed exponential cultures (shake flask, dashed line) and the mixed aged cultures (shake flask, solid line), error bars represent standard deviation of biological triplicates; nomenclature as in main manuscript

References

Biechele, P., Busse, C., Solle, D., Scheper, T., and Reardon, K. (2015). Sensor systems for bioprocess monitoring. *Engineering in Life Sciences*, 15(5):469–488.

Möller, J., Bhat, K., Riecken, K., Pörtner, R., Zeng, A.-P., and Jandt, U. (2019). Process-induced cell cycle oscillations in cho cultures: online monitoring and model-based investigation. *Biotechnology and Bioengineering*, (Accepted manuscript).

Nagai, T., Ibata, K., Park, E. S., Kubota, M., Mikoshiba, K., and Miyawaki, A. (2002). A variant of yellow fluorescent protein with fast and efficient maturation for cell-biological applications. *Nature biotechnology*, 20(1):87.

Rizzo, M. A., Springer, G. H., Granada, B., and Piston, D. W. (2004). An improved cyan fluorescent protein variant useful for fret. *Nature biotechnology*, 22(4):445.

Shaner, N. C., Campbell, R. E., Steinbach, P. A., Giepmans, B. N., Palmer, A. E., and Tsien, R. Y. (2004). Improved monomeric red, orange and yellow fluorescent proteins derived from discosoma sp. red fluorescent protein. *Nature biotechnology*, 22(12):1567.

Shaner, N. C., Lin, M. Z., McKeown, M. R., Steinbach, P. A., Hazelwood, K. L., Davidson, M. W., and Tsien, R. Y. (2008). Improving the photostability of bright monomeric orange and red fluorescent proteins. *Nature methods*, 5(6):545.

Subach, O. M., Cranfill, P. J., Davidson, M. W., and Verkhusha, V. V. (2011). An enhanced monomeric blue fluorescent protein with the high chemical stability of the chromophore. *PloS one*, 6(12):e28674.
